# Thermal treatment of water-soluble particles formed by compounds composed of carbon nanobelts and C_60_ molecules

**DOI:** 10.1038/s41598-023-45840-7

**Published:** 2023-10-28

**Authors:** Shunji Kurosu, Sayaca Hata, Tomofumi Ukai, Yuta Mashiko, Sieun Choi, Takanobu Minakawa, Yuri Tanuma, Toru Maekawa

**Affiliations:** 1 Bio-Nano Electronics Research Centre, Toyo University, 2100, Kujirai, Kawagoe, 350-8585 Japan; 2 Graduate School of Interdisciplinary New Science, Toyo University, 2100, Kujirai, Kawagoe, 350-8585 Japan; 3 Graduate School of Science and Engineering, Toyo University, 2100, Kujirai, Kawagoe, 350-8585 Japan; 4 Jožef Stefan Institute, Jamova 39, SI-1000 Ljubljana, Slovenia

**Keywords:** Materials science, Nanoscience and technology

## Abstract

It was previously shown that spherical particles are self-assembled by compounds composed of C_60_-(6,6)CNB-C_60_, where CNB stands for “carbon nanobelt”, by mixing two individual solutions of C_60_ and (6,6)CNB molecules dissolved in 1,2-dichlorobenzene at room temperature. The particles are monodisperse in water thanks to their high absolute value of the zeta potential in water. In this report, we investigate the effect of thermal treatment of the particles on some changes in the physical properties and structures. We find that the particles become electrically conductive after thermal treatment at 600 °C for 1 h. We suppose that the change in the electrical characteristics might have been caused by the structural change of (6,6)CNBs into opened-up ribbons composed of fused benzene rings, which construct networks supported by C_60_ molecules in the particles, judging by the change in the absorption and mass spectra of the particles after thermal treatment and analysis of a possible change in the structure of C_60_-(6,6)CNB-C_60_ based on quantum chemical calculations employing the PM6 method, with which it is known that nanostructures such as carbon nanotubes (CNTs) and (6,6)CNBs can be correctly estimated.

## Introduction

**Figure 1:**
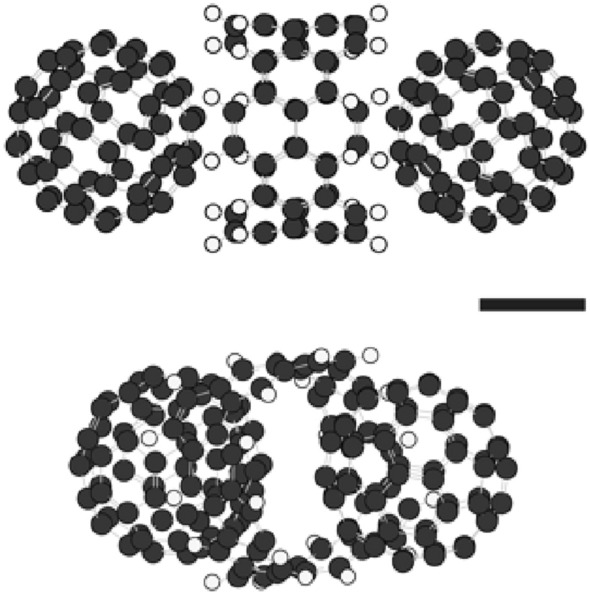
Compound composed of two C_60_ molecules and one (6,6)CNB. Two views from different angles are shown. Gray and white circles, respectively, represent carbon and hydrogen atoms. Spherical particles were formed by the compounds^16^. The formation energy $E \equiv E\left( {{\text{C}}_{{60}} {-}{\text{CNB}}{-}{\text{C}}_{{60}} } \right) - E\left( {{\text{CNB}}} \right) - 2 \times E\left( {{\text{C}}_{{60}} } \right) = - ~0.19\,{\text{eV}}$. The scale bar represents 0.4 nm.

A carbon nanobelt (CNB) is a loop of fused benzene rings and various types of CNBs have been successfully synthesised in recent years^1–7^. The structures of CNBs, structural transitions and the effect of external electric fields on the structures of CNBs have been theoretically and numerically analysed^8–11^ and several devices, in which CNBs are used, have been designed and developed^12–14^. A C_60_ molecule is one form of fullerenes composed of 60 carbon atoms^15^. The interaction between CNBs and C_60_ molecules has not yet been intensively studied^16^ although the structure formed by cycloparaphenylene (CPP, carbon nanorings) and C_60_ molecules is now well understood^17–21^. We recently found that spherical particles of a uniform diameter are self-assembled at room temperature by mixing two individual solutions of (6,6)CNBs and C_60_ molecules dissolved in 1,2-dichlorobenzene^16^. The particles are monodisperse in water thanks to their high absolute value of the zeta potential. The particles are in fact formed by charge-transfer compounds; C_60_-(6,6)CNB-C_60_ (see Fig. 1). In this study, we investigate the effect of thermal treatment of the particles on some changes in the physical properties and internal structures. We find that the particles are still monodisperse in water and become electrically conductive after thermal treatment at 600 °C for 1 h. Characterisations of the particles before and after thermal treatment are carried out by Zetasiser, scanning and transmission electron microscopy (SEM and TEM), selected area electron and X-ray diffractometry (SAED and XRD), and absorption and time-of-flight mass spectroscopy (TOF–MS). We suppose that the change in the electric conductivity might have been caused by the structural change of (6,6)CNBs into opened-up ribbons of fused benzene rings, which construct networks supported by C_60_ molecules in the particles.

## Results and discussion

**Figure 2:**
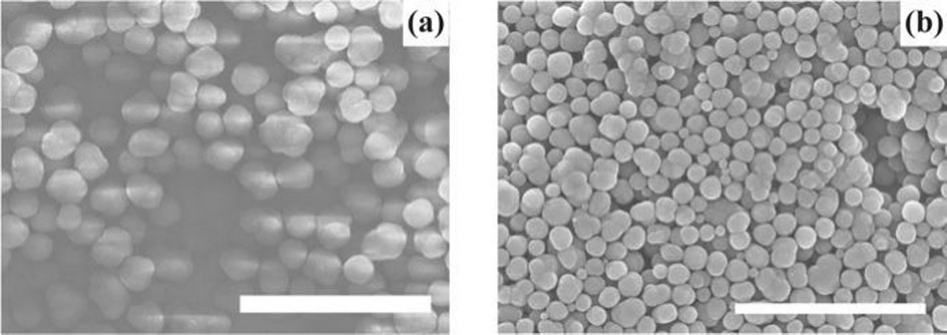
SEM images of particles formed by compounds composed of (6,6)CNBs and C_60_ molecules. The particles were synthesised by mixing two solutions of (6,6)CNBs and C_60_ molecules dissolved in 1,2-dichlorobenzene. The molar concentrations of (6,6)CNBs and C_60_ molecules were 0.35 and 0.70 µmol ml^−1^ after the mixture of two solutions. The particles were thermally treated at 600 °C for 1 h. The scale bars represent 5 µm. (**a**) Particles without thermal treatment. A charge-up phenomenon occurred. (**b**) Particles after thermal treatment at 600 °C. There was no charge-up.

We synthesised spherical particles of a uniform diameter in 1,2-dichlorobenzene by mixing two individual solutions of (6,6)CNBs and C_60_ molecules dissolved in 1,2-dichlorobenzene following the previous methodology^16^, where the molar concentrations of (6,6)CNBs and C_60_ molecules were, respectively, set at 0.7 and 1.4 µmol ml^-1^. The particles were then thermally treated at 600 °C for 1 h in a thermogravimetric (TG) analyser. SEM images of the particles before and after thermal treatment are shown in Fig. 2, where Fig. 2a,b, respectively, represent the particles without thermal treatment and those after thermal treatment at 600 °C. A charge-up (charge accumulation) phenomenon was observed on the surface of the particles before thermal treatment (see Fig. 2a), whereas there was no charge accumulation on the surface of the particles after thermal treatment at 600 °C (Fig. 2b). This suggests that the electrical characteristics of the particles might have been changed by thermal treatment of the particles at 600 °C, which will be discussed later.

The diameter of a particle, and the hydrodynamic diameter and zeta potential of a particle dispersed in distilled water before and after thermal treatment at 600 °C are shown in Table 1. The diameter of a particle slightly decreased after thermal treatment. The decrease in the diameter of a particle coincides with the decrease in the weight of a particle during thermal treatment; that is, ${{{w}_{2}/w}_{1}\approx \left({{d}_{2}/d}_{1}\right)}^{3}$, where $w$ and $d$ are the weight and diameter of a particle and subscripts 1 and 2 represent before and after thermal treatment (see Fig. S1 in the Supplementary Information for the time variation of the temperature and the weight of the particles during thermal treatment). Note that there was no significant weight loss during thermal treatment of the particles at 300 °C for 1 h^16^ and therefore it is supposed that the present weight loss was not caused by the evaporation of the solvent captured in the particles. Even after thermal treatment, the absolute value of the zeta potential of a particle dispersed in distilled water was as high as 35.4 mV and as a result, the particles were monodisperse in water. The particles finally precipitated in distilled water due to gravity, but once the suspension having been shaken, the particles monodispersed again thanks to the high absolute value of the zeta potential in water (see Fig. S3 and Video S1 in the Supplementary Information, respectively, for the time variation of the turbidity of the suspension and for the redispersion of the particles after a shake of the suspension).

**Table 1:** Diameter of a particle, and the hydrodynamic diameter and zeta potential of a particle dispersed in distilled water before and after thermal treatment.

	Diameter (nm)	Hydrodynamic diameter (nm)	Zeta potential (mV)
Before thermal treatment	(5.15 ± 0.62) × 10^2^	(1.30 ± 0.09) × 10^3^	− 38.8 ± 0.7
After thermal treatment at 600 °C for 1 h	(4.44 ± 0.63) × 10^2^	(0.86 ± 0.02) × 10^3^	− 35.4 ± 1.0

**Figure 3:**
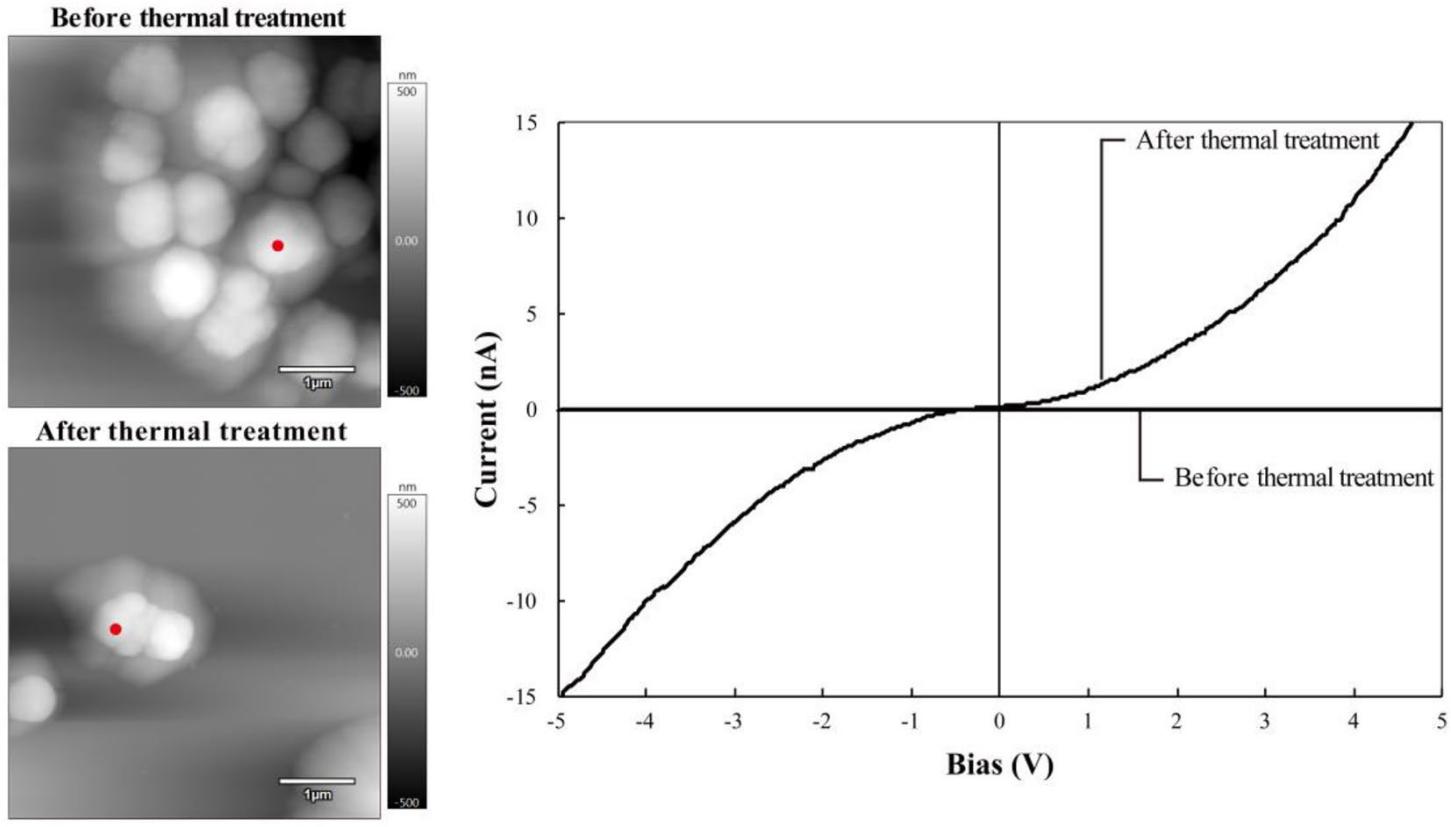
I–V characteristics of the particles before and after thermal treatment. There was no clear hysteresis in the I–V curves and the current was averaged over five consecutive measurements. AFM images of particles are also shown on the left, where the scale bars represent 1.0 µm. The red spots on particles represent the points on which the conductive probe was imposed. The particles were insulators before thermal treatment, whereas they became conductive after thermal treatment.

The current–voltage (I–V) characteristics of the particles, which were measured using an atomic force microscope (AFM)^22,23^, are shown in Fig. 3, where the current is averaged over five consecutive measurements, noting that there was no clear hysteresis in the I–V curves, and AFM images of the particles are also shown, the red spots on the surface of the particles representing the points contacted by a conductive diamond probe. As is clearly shown, there was no current through the particle before thermal treatment, whereas the particle became electrically conductive after thermal treatment at 600 °C. Let us make a rough estimate of the conductivity of the particles after thermal treatment. The radius of the tip of the conductive diamond probe, $r$, the electric potential difference, $\Delta \phi$, the electric current, $I$, and the diameter of a particle, $d$, being 10 nm, 5 V, 15 nA and 440 nm, the conductivity of the particle, $\sigma =i/E$, where $i=I/\left(\pi {r}^{2}\right)$ and $E=\Delta \phi /d$, are the current density and electric field, would be of the order of 5 S m^−1^. Note that the charge-up phenomenon was still observed with SEM microscopy in the case of the particles after thermal treatment at 500 °C for 1 h (Fig. S3 in the Supplementary Information for an SEM image of particles after thermal treatment at 500 °C) and it is therefore supposed that the insulator-conductor transition occurred between 500 and 600 °C.

**Figure 4:**
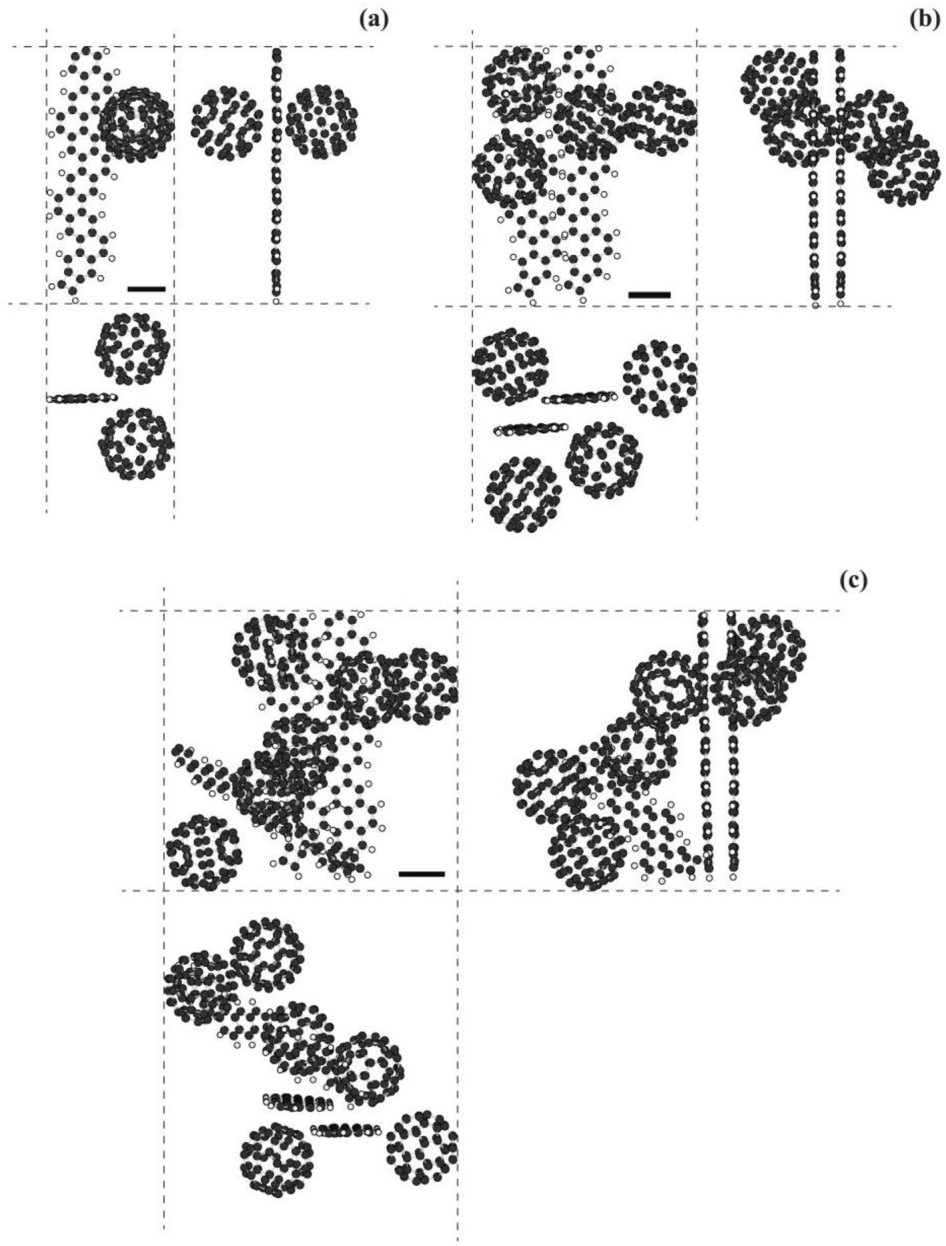
Structures of CNR/C_60_ compounds calculated with PM6. (**a**) CNR/C_60_ compound composed of two C_60_ molecules and one CNR. The formation energy $E \equiv E\left( {{\text{C}}_{{60}} {-}{\text{CNR}}{-}{\text{C}}_{{60}} } \right) - E\left( {{\text{CNR}}} \right) - 2 \times E\left( {{\text{C}}_{{60}} } \right) = - ~0.18~{\text{eV~and~}} - ~0.0011~{\text{eV}}/{\text{atom}}$. The decomposition energy $E \equiv E\left( {{\text{C}}_{{60}} {-}{\text{CNR}}{-}{\text{C}}_{{60}} } \right) - E\left( {{\text{C}}_{{60}} {-}{\text{CNB}}{-}{\text{C}}_{{60}} } \right) = 3.14~{\text{eV~and~}}0.019~{\text{eV}}/{\text{atom}}$. (**b**) CNR/C_60_ compounds composed of four C_60_ molecules and two CNRs. The formation energy $E \equiv E\left( {2 \times ({\text{C}}_{{60}} {-}{\text{CNR}}{-}{\text{C}}_{{60}} )} \right) - 2 \times E\left( {{\text{CNR}}} \right) - 4 \times E\left( {{\text{C}}_{{60}} } \right) = - ~0.75~{\text{eV~and~}} - ~0.0022~{\text{eV}}/{\text{atom}}$. The decomposition energy $E \equiv E\left( {2 \times ({\text{C}}_{{60}} {-}{\text{CNR}}{-}{\text{C}}_{{60}} )} \right) - E\left( {2 \times ({\text{C}}_{{60}} {-}{\text{CNB}}{-}{\text{C}}_{{60}} )} \right) = 6.07~{\text{eV~and}}~0.018~{\text{eV}}/{\text{atom}}$. (**c**) CNR/C_60_ compounds composed of six C_60_ molecules and three CNRs. The formation energy $E \equiv E\left( {3 \times ({\text{C}}_{{60}} {-}{\text{CNR}}{-}{\text{C}}_{{60}} )} \right) - 3 \times E\left( {{\text{CNR}}} \right) - 6 \times E\left( {{\text{C}}_{{60}} } \right) = - ~1.14~{\text{eV~and~}} - ~0.0023~{\text{eV}}/{\text{atom}}$. The decomposition energy $E \equiv E\left( {3 \times ({\text{C}}_{{60}} {-}{\text{CNR}}{-}{\text{C}}_{{60}} )} \right) - E\left( {3 \times ({\text{C}}_{{60}} {-}{\text{CNB}}{-}{\text{C}}_{{60}} )} \right) = 9.05~{\text{eV~and~}}0.018~{\text{eV}}/{\text{atom}}$. The scale bars represent 0.4 nm.

As explained above, the particles were originally formed by compounds composed of one (6,6)CNB and two C_60_ molecules; that is, C_60_-(6,6)CNB-C_60_, (see Fig. 1), and therefore, it is supposed that the alteration in the electrical characteristics after thermal treatment at 600 °C might have been caused by some structural change of the compounds in the particles. However, there were no dramatic changes in the TEM images, and SAED and XRD patterns (see Figs. S4 and S5 in the Supplementary Information for the TEM images/SAED patterns and XRD patterns before and after thermal treatment at 600 °C). The absorption spectra of particles before and after thermal treatment are shown in Fig. S6 in the Supplementary Information. The wavelength corresponding to the absorption peak was 204 nm in the case of the particles without thermal treatment, whereas the peak disappeared after thermal treatment at 600 °C, noting that the particles before thermal treatment were composed of compounds; C_60_-(6,6)CNB-C_60_, in which C_60_ and (6,6)CNB are bonded via charge transfer, and the absorption wavelength corresponds to charge transition^16^. It is, therefore, supposed that the structure of the compounds; C_60_-(6,6)CNB-C_60_, might have been changed after thermal treatment. According to the TOF–MS of the particles, (6,6)CNBs and C_60_ molecules were detected before thermal treatment, whereas there was no (6,6)CNBs observed, although C_60_ was detected, after thermal treatment at 600 °C (see Fig. S7 in the Supplementary Information for the TOF–MS), which suggests that (6,6)CNB might have been broken or dissociated during thermal treatment at 600 °C for 1 h. We carried out quantum chemical calculations concerning a structural change from a (6,6)CNB to a carbon nano ribbon (CNR)^24–28^ disconnecting a pair of fused benzene rings by semi-empirical PM6^29^. It is known that the structures of carbon nanostructures such as carbon nanotubes (CNTs) and CNBs can be correctly estimated with the PM6 method^16,30–32^. In the present study, the most stable structure obtained with PM6 among opened-up ribbons was the one terminated with a square, which coincided with the structure obtained with the density functional theory (DFT) (see Fig. S8). The details of the structures of the opened-up ribbon obtained with PM6 and DFT are also summarised in Fig. S8. The structures formed by ribbon/C_60_ complexes are shown in Fig. 4. The structures formed by compounds; C_60_-(6,6)CNB-C_60_, which corresponds to those before thermal treatment, are also shown in Fig. S9. The configuration formed by one ribbon and two C_60_ molecules is shown in Fig. 4a. Some part of the ribbon is positioned between two C_60_ molecules. As the number of C_60_ and CNBs molecules increases, the configurations become more complicated (Fig. 4b,c). Those compounds were not regularly located in the particles both before and after thermal treatment (see Figs. 4 and S9), which explains why there were no dramatic changes in the TEM images, and SAED and XRD patterns (Figs. S4 and S5). It is supposed that the in-ribbon electric conductivity is high in the longitudinal direction^28,33–35^, whereas the conductivity across two layers of ribbons is low^33–35^. The overall conductivity is determined by both in-ribbon and cross-ribbons electron transports and in the present case, the conductivity was of the order of 5 S m^-1^. We suppose that (6,6)CNBs may be changed to opened-up ribbons as mentioned above and particles are filled with ribbon/C_60_ complexes, the ribbons constructing networks supported by C_60_ molecules during thermal treatment, which explains the disappearance of charge accumulation on the surface of the particles (see Fig. 2b), the appearance of conductivity of the particles^33–41^ (Fig. 3), the disappearance of the peak in the absorption spectrum (Fig. S6) and the disappearance of (6,6)CNBs in the mass spectrum (Fig. S7) after thermal treatment of particles at 600 °C.

Networks of ribbons can be constructed in particles via pyrolysis as shown in the present study, but we suppose that networks may also be created on the surface of particles via photolysis with irradiation of ultraviolet (UV) laser beams. It would also be possible to attract compounds; C_60_-(6,6)CNB-C_60_, to an anode in a dc electric field, noting that C_60_ is negatively charged in the compound^16^, and then the (6,6)CNBs would be opened up via either pyrolytic or photolytic treatment of the compounds (see Fig. 4a), which may lead to the development of nano circuits technology. The particles are monodisperse in water even after thermal treatment, which means that the particles can be used as conductive colloidal ones.

## Conclusion

We produced water-soluble particles of a uniform diameter composed of compounds; C_60_-(6,6)CNB-C_60_, at room temperature by mixing two individual solutions of carbon nanobelts and C_60_ molecules dissolved in 1,2-dichlorobenzene. We then found that the particles become electrically conductive after thermal treatment of the particles at 600 °C for 1 h. The mechanism of the change in the electrical characteristics after thermal treatment is an open question, but we suppose that the change in the electrical conductivity of particles after thermal treatment might have been caused by the structural change of carbon nanobelts into opened-up ribbons of fused benzene rings, which construct networks assisted by C_60_ molecules. The creation of nano/micro structures and materials using basic carbon nano units such as CNB, CPP and fullerenes such as C_60_, C_70_ and C_59_N^42,43^ is our future task.

## Methods

### Synthetic procedure of particles^16^

- (6,6)CNBs (Tokyo Chemical Industry Co. Ltd.) and C_60_ molecules (Kanto Chemical Co. Inc.) were individually dissolved in 1,2-dichlorobenzene. The molar concentrations of (6,6)CNBs and C_60_ molecules dissolved in 1,2-dichlorobenzene were, respectively, set at 0.7 and 1.4 µmol ml^-1^.
- Those two solutions were mixed at room temperature, noting that the molar concentrations of (6,6)CNBs and C_60_ molecules dissolved in 1,2-dichlorobenzene became 0.35 and 0.7 µmol ml^-1^ after the mixture of the solutions.
- The mixed solution was left still for 1 week in a dark box at room temperature.
- 1,2-dichlorobenzene was then replaced by ethanol, followed by sonication and centrifugation twice.
- The particles separated by centrifugation were dispersed in distilled water, followed by sonication.

### Thermal treatment of particles

- The above particles were placed in a thermogravimetric (TG) analyser (DTG-60, Shimadzu Corp.).
- The temperature was raised up to 600 °C at a rate of 15.9 K min^-1^ and then kept at 600 °C for 1 h with the flow of N_2_ gas. Then, the temperature was decreased naturally down to room temperature. The time variation of the temperature and the weight of the particles was recorded. The weight was calibrated with a precision scale (Excellence plus XP56, Mettler-Toledo International Inc.).

### Observation and characterisation of the particles

**Figure 5:**
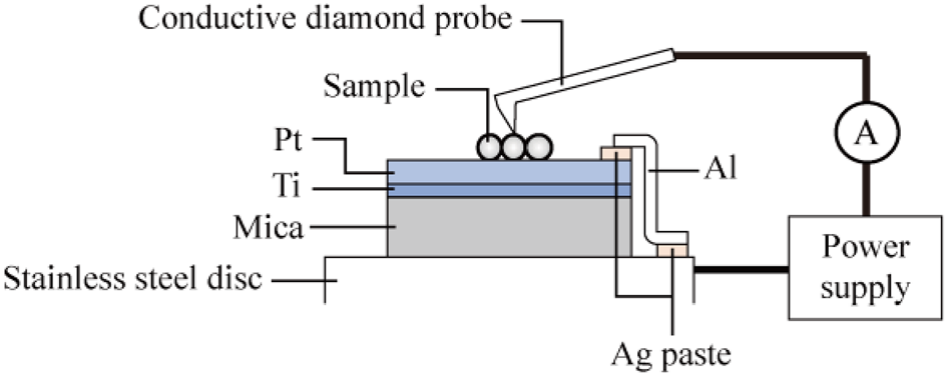
Outline of the measurement system of the I–V characteristics of particles. Particles were placed on a platinum film and the current is measured using a diamond conductive probe imposing a force of 10 nN onto the top of a particle.
- A droplet of the suspension of particles dispersed in ethanol was dropped onto a silicon-low background sample holder (M00016288, Rigaku Corp.) and X-ray diffractometric characterisation of the particles was carried out by X-ray diffractometry (SmartLab, Rigaku Corp.).
- The absorption spectrum by the particles dispersed in ethanol was measured by ultraviolet-visible (UV-Vis) spectroscopy (DU730, Beckman Coulter Inc.).
- The molecular weight of molecules forming the particles was measured with TOF-MS (autoflex2, Bruker Co.).

- The structures of the particles were observed by SEM (SU8030, Hitachi Ltd.) and TEM (2200FS, JEOL Ltd. and ARM200F, JEOL Ltd.), where SAED patterns were also obtained.
- The size of the particles was measured, targeting at 100 particles from the SEM images.
- The hydrodynamic diameter and zeta potential of the particles dispersed in distilled water were measured by Zetasizer (Nano-ZS, Malvern Panalytical Ltd.).
- The precipitation process of the particles dispersed in distilled water was observed and recorded on the hard disc of a computer. The intensity of the transmitted light of 700 nm wavelength through the whole solution confined in a glass container was measured with a spectral photometer (U-3500 Spectrophotometer, Hitachi High-Tech Co.) and the turbidity, which was defined by $\left(1-{I}_{trans}/{I}_{in}\right)\times 100 \%$, where ${I}_{in}$ and ${I}_{trans}$ are, respectively, the intensities of the incident and transmitted light, was obtained.
- The current–voltage (I–V) characteristics of the particles were measured using an atomic force microscope (AFM) (Cypher, Oxford Instruments PLC.). First, a droplet of the suspension of the particles dispersed in ethanol was dropped onto the surface of a substrate, which was composed of platinum and titanium films deposited on a mica base (see Fig. 5) and then ethanol was evaporated naturally. The substrate, on the surface of which the particles were placed, was set on a metal disc. The metal disc and the platinum film were connected by an aluminium foil and silver paste. The surface morphology of the particles was measured by the AFM and then the I-V curves were obtained using a conductive diamond probe^21,22^ (AD-2.8-AS, Adama Innovations Ltd.), imposing a force of 10 nN onto the top of the particles. The electric voltage was changed as follows; 0 → 10 → 0 → - 10 → 0 V, which was repeated five times, and the average current was calculated. The measurement range of the current was from – 20 to 20 nA.

### Quantum chemical simulations

The structures of an opened-up CNR, and CNR/C_60_ and CNB/ C_60_ compounds were calculated with PM6^29^ (Gaussian 16 Revision A.03, Gaussian Inc).

The decomposition energy and formation energy were defined as follows.

Decomposition energy of a CNB into CNR:

$$E \equiv E\left( {{\text{CNR}}} \right) - E\left( {{\text{CNB}}} \right)$$

Decomposition energy of compounds; C_60_-CNB-C_60_, into C_60_-CNR-C_60_:

$$E \equiv E\left( {n \times ({\text{C}}_{{60}} {-}{\text{CNR}}{-}{\text{C}}_{{60}} )} \right) - E\left( {n \times ({\text{C}}_{{60}} {-}{\text{CNB}}{-}{\text{C}}_{{60}} )} \right)$$

Formation energy of compounds; C_60_-CNB-C_60_:

$$E \equiv E\left( {n \times ({\text{C}}_{{60}} {-}{\text{CNB}}{-}{\text{C}}_{{60}} )} \right) - n \times E\left( {{\text{CNB}}} \right) - 2n \times E\left( {{\text{C}}_{{60}} } \right)$$

Formation energy of compounds; C_60_-CNR-C_60_:

$$E \equiv E\left( {n \times ({\text{C}}_{{60}} {-}{\text{CNR}}{-}{\text{C}}_{{60}} )} \right) - n \times E\left( {{\text{CNR}}} \right) - 2n \times E\left( {{\text{C}}_{{60}} } \right)$$

In the present study, *n* = 1, 2, 3.

### Supplementary Information

Supplementary Figures.

Supplementary Video 1.

## Data Availability

All of the data supporting this work are available from the corresponding author upon reasonable request.

## References

[1] Povie G, Segawa Y, Nishihara T, Miyauchi Y, Itami K (2017). Synthesis of a carbon nanobelt. Science.

[2] Wegner HA (2017). On the way to carbon nanotubes: The first synthesis of an aromatic nanobelt. Angew. Chem. Int. Ed..

[3] Lu X, Wu J (2017). After 60 years of feforts: The chemical synthesis of a carbon nanobelt. Chem.

[4] Wang J, Miao Q (2019). A tetraazapentacene-pyrene belt: Toward synthesis of N-doped zigzag carbon nanobelts. Org. Lett..

[5] Xue S, Kuzuhara D, Aratani N, Yamada H (2019). Synthesis of a porphyrin(2.1.2.1.) nanobelt and its ability to bind fullerene. Org. Lett..

[6] Cheung KY, Gui S, Deng C, Liang H, Xia Z, Liu Z, Chi L, Miao Q (2019). Synthesis of armchair and chiral carbon nanobelts. Chem.

[7] Bergman HM, Kiel GR, Handford RC, Liu Y, Tilley TD (2021). Scalable, divergent synthesis of a high aspect ratio carbon nanobelt. J. Am. Chem. Soc..

[8] Miranda WDSA, Frazão NF, Moreira E, Azevedo DL (2022). Penta-belt: A new carbon nanobelt. J. Mol. Struct..

[9] Tanuma Y, Dunk P, Maekawa T, Ewels C (2022). Chain and fullerene formation during hydrogen loss and reconstruction in non-planar polyaromatic hydrocarbons. Nanomaterials.

[10] Lu C, Jiang F, Wang J (2022). [6,6]CNB with controllable external electric field deformation: A theoretical study of the structure-function relationship. Mater. Res. Express.

[11] Tanuma Y, Maekawa T, Ewels C (2021). Methodological investigation for hydrogen addition to small cage carbon fullerenes. Crystals.

[12] Yu X, Wang M, Gagnoud A, Fautrelle Y, Moreau R, Li X (2017). Fabrication and electrochemical properties of a graphene-enhanced hierarchical porous network of Fe_3_O_4_/carbon nanobelts. Electrochim. Acta.

[13] Ma C, Cao E, Dirican M, Subjalearndee N, Cheng H, Li J, Song Y, Shi J, Zhang X (2021). Fabrication, structure and supercapacitance of flexible porous carbon nanobelt webs with enhanced inter-fiber connection. Appl. Surf. Sci..

[14] Cheng Y, Zhang Y, Jiang H, Dong X, Meng C, Kou Z (2020). Coupled cobalt silicate nanobelt-on-nanobelt hierarchy structure with reduced graphene oxide for enhanced supercapacitive performance. J. Power Sources.

[15] Kroto HW, Heath JR, O’Brien SC, Curl RF, Smalley RE (1985). C_60_: Buckminsterfullerene. Nature.

[16] Choi S, Kurosu S, Mashiko Y, Minakawa T, Maekawa T (2022). Room temperature synthesis of water-soluble spherical particles of a uniform diameter composed of carbon nanobelts and C_60_ molecules. Sci. Rep..

[17] Xia J, Bacon JW, Jasti R (2012). Gram-scale synthesis and crystal structures of [8]- and [10]CPP, and the solid-state structure of C_60_@[10]CPP. Chem. Sci..

[18] González-Veloso I, Cabaleiro-Lago EM, Rodríguez-Otero J (2018). Fullerene size controls the selective complexation of [11]CPP with pristine and endohedral fullerenes. Phys. Chem. Chem. Phys..

[19] Evans PJ, Zakharov LN, Jasti R (2019). Synthesis of carbon nanohoops containing thermally stable cis azobenzene. J. Photochem. Photobiol..

[20] González-Veloso I, Rodríguez-Otero J, Cabaleiro-Lago EM (2019). Endohedral alkali cations promote charge transfer transitions in complexes of C60 with [10]cycloparaphenylenes. Phys. Chem. Chem. Phys..

[21] Zhang J, Zhao C, Meng H, Nie M, Li Q, Xiang J, Zhang Z, Wang C, Wang T (2020). Size-selective encapsulation of metallofullerenes by [12]cycloparaphenylene and dissociation using metal-organic framework. Carbon.

[22] Jiang L, Weber J, Puglisi FM, Pavan P, Larcher L, Frammelsberger W, Benstetter G, Lanza M (2019). Understanding current instabilities in conductive atomic force microscopy. Materials.

[23] Sundqvist P, Garcia-Vidal FJ, Flores F, Moreno-Moreno M, Gómez-Navarro C, Bunch JS, Gómez-Herrero J (2007). Voltage and length-dependent phase diagram of the electronic transport in carbon nanotubes. Nano Lett..

[24] Cançad LG, Pimenta MA, Neves BRA, Medeiros-Ribeiro G, Enoki T, Kobayashi Y, Takai K, Fukui K, Dresselhaus MS, Saito R, Jorio A (2004). Anisotropy of the Raman spectra of nanographite ribbons. Phys. Rev. Lett..

[25] Terrés B, Reichardt S, Neumann C, Watanabe K, Taniguchi T, Stampfer C (2014). Raman spectroscopy on mechanically exfoliated pristine graphene ribbons. Phys. Status Solidi B.

[26] Tsetseris T, Pantelides ST (2011). Graphene nano-ribbon formation through hydrogen-induced unzipping of carbon nanotubes. Appl. Phys. Lett..

[27] Fitzgibbons TC, Guthrie M, Xu E, Crespi VH, Davidowski SK, Cody GD, Alem N, Badding JV (2015). Benzene-derived carbon nanothreads. Nat. Mater..

[28] Huang YC, Chang CP, Lin MF (2008). Electric-field induced modification of electronic properties of few-layer graphene nanoribbons. J. Appl. Phys..

[29] Stewart JJP (2007). Optimization of parameters for semiempirical methods V: Modification of NDDO approximations and application to 70 elements. J. Mol. Model..

[30] Chang X, Lin S, Wang G, Shang C, Wang Z, Liu K, Fang Y, Stang PJ (2020). Self-assembled perylene bisimide-cored trigonal prism as an electron-deficient host for C_60_ and C_70_ driven by “Like dissolves like”. J. Am. Chem. Soc..

[31] Conley KM, Whiteheada MA, van de Vena TGM (2019). Linear growth of self-assembled alternating oligopeptide nanotubes with self-locking building blocks. Mol. Simul..

[32] Ding F (2005). Theoretical study of the stability of defects in single-walled carbon nanotubes as a function of their distance from the nanotube end. Phys. Rev. B.

[33] Chen J, Gao X (2019). Directional dependence of electrical and thermal properties in graphene nanoplatelet- based composite materials. Results Phys..

[34] Celzard A, Marêché JF, Furdin G, Puricelli S (2000). Electrical conductivity of anisotropic expanded graphite-based monoliths. J. Phys. D Appl. Phys..

[35] Cermak M, Perez N, Collins M, Bahrami M (2020). Material properties and structure of natural graphite sheet. Sci. Rep..

[36] Pietronero L, Strassler S, Zeller HR, Rice MJ (1980). Electrical conductivity of a graphite layer. Phys. Rev. B.

[37] Chen SC, Chang CP, Lee CH, Lin MF (2010). Tuning of electronic properties of nanographene ribbons by a spatially modulated electric field. J. Appl. Phys..

[38] Kumar SB, Fujita T, Liang G (2011). Conductance modulation in graphene nanoribbon under transverse asymmetric electric potential. J. Appl. Phys..

[39] Lin TS, Lin MF, Chang SC, Lin TC (2012). Electronic properties of bearded graphene nanoribbons. J. Phys. Chem. Solids.

[40] Li TS, Lin MF, Lin CY, Chang SC, Yang SP (2013). Electronic properties of curved graphene nanoribbons. Synth. Met..

[41] Seenithurai S, Chai J-D (2021). Electronic properties of carbon nanobelts predicted by thermally-assisted-occupation DFT. Nanomaterials.

[42] Rio J, Beeck S, Rotas G, Ahles S, Jacquemin D, Tagmatarchis N, Ewels C, Wegner HA (2018). Electronic communication between two [10]cycloparaphenylenes and bis(azafullerene) (C_59_N)_2_ induced by cooperative complexation. Angew. Chem. Int. Ed..

[43] Tanuma Y, Stergiou A, Bobnar AB, Gaboardi M, Rio J, Volkman J, Wegner HA, Tagmatarchis N, Ewels CP, Arčon D (2021). Robust coherent spin centers from stable azafullerene radicals entrapped in cycloparaphenylene rings. Nanoscale.

