# Supplementary material for: Thermal treatment of water-soluble particles formed by compounds composed of carbon nanobelts and C60 molecules
Source: Sci Rep. 2023 Oct 28;13:18480. doi: 10.1038/s41598-023-45840-7 (PMC10613224; doi:10.1038/s41598-023-45840-7)
Supplement: Supplementary file 1 — Supplementary Figures. [file 41598_2023_45840_MOESM1_ESM.docx]

**Supplementary Information**

**Thermal treatment of water-soluble particles formed by**

**compounds composed of carbon nanobelts and C_60_ molecules**

Shunji Kurosu^1,2^, Sayaca Hata^3^, Tomofumi Ukai^1,2^, Yuta Mashiko^2^,

Sieun Choi^2^, Takanobu Minakawa^2^, Yuri Tanuma^1,4^ and Toru Maekawa^1,2,^*

^1^ Bio-Nano Electronics Research Centre, Toyo University, 2100, Kujirai, Kawagoe, Japan

^2^ Graduate School of Interdisciplinary New Science, Toyo University, 2100, Kujirai, Kawagoe, Japan

^3^ Graduate School of Science and Engineering, Toyo University, 2100, Kujirai, Kawagoe, Japan

^4^ Jožef Stefan Institute, Jamova 39, SI-1000 Ljubljana, Slovenia

* Corresponding author: [maekawa@toyo.jp](mailto:maekawa@toyo.jp)

--------------------------------------------------------------------

**
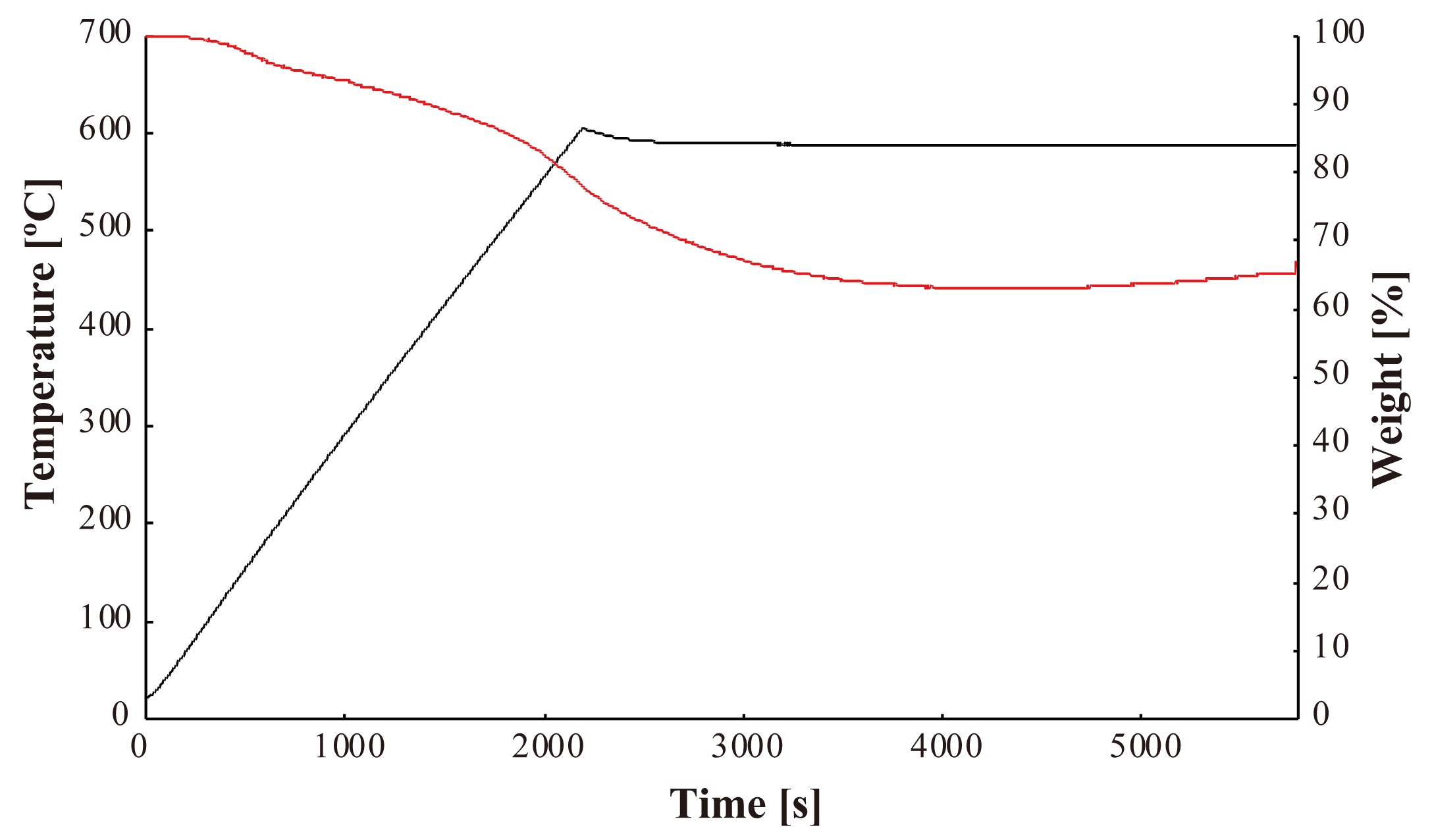
**


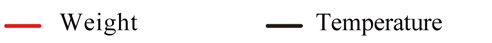
**Fig. S1 Time variation of the weight of a particle and the temperature during thermal treatment.** The temperature was raised up to 600 °C at a rate of 15.9 K min^-1^ and kept at 600 °C for 1 h with the flow of N_2_ gas. Then, the temperature was decreased naturally down to room temperature. The weight was calibrated with a precision scale.

**
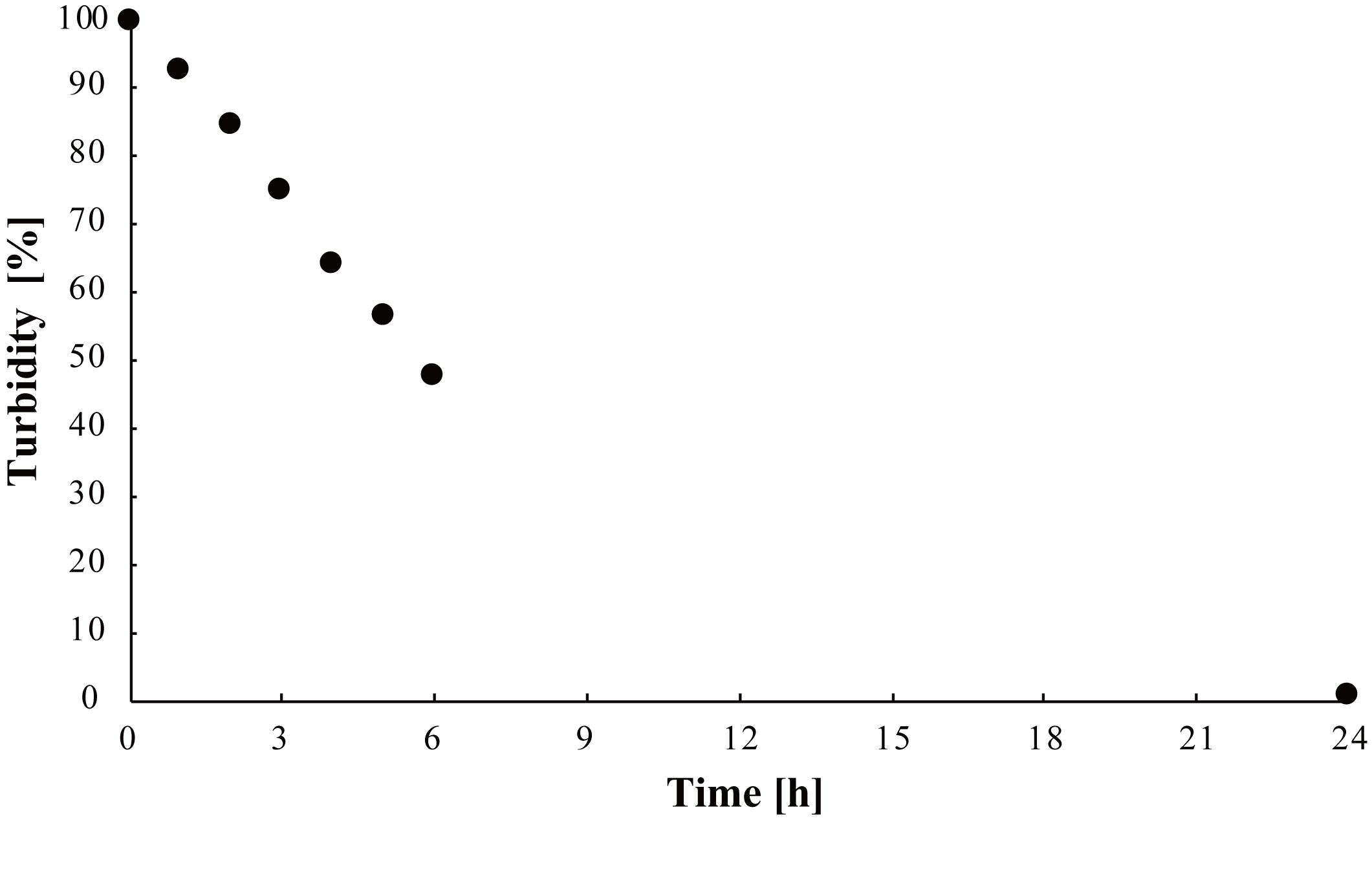
**

**Fig. S2 Time variation of the turbidity of the solution of particles dispersed in distilled water.** The intensity of the transmitted light of 700 nm wavelength through the whole solution confined in a glass container was measured. The turbidity was defined as $\left( 1-{I_{trans}}/{I_{in}} \right)\times100 \%$, where $I_{in}$ and $I_{trans}$ are, respectively, the intensities of the incident and transmitted light.


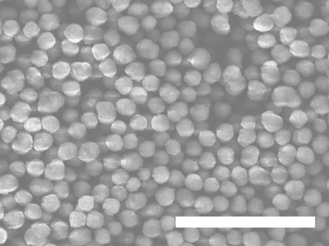


**Fig. S3 SEM image of particles formed by compounds composed of (6,6)CNBs and C_60_ molecules.** The particles were thermally treated at 500 °C for 1 h. The scale bars represent 5 µm. A charge-up phenomenon occurred.


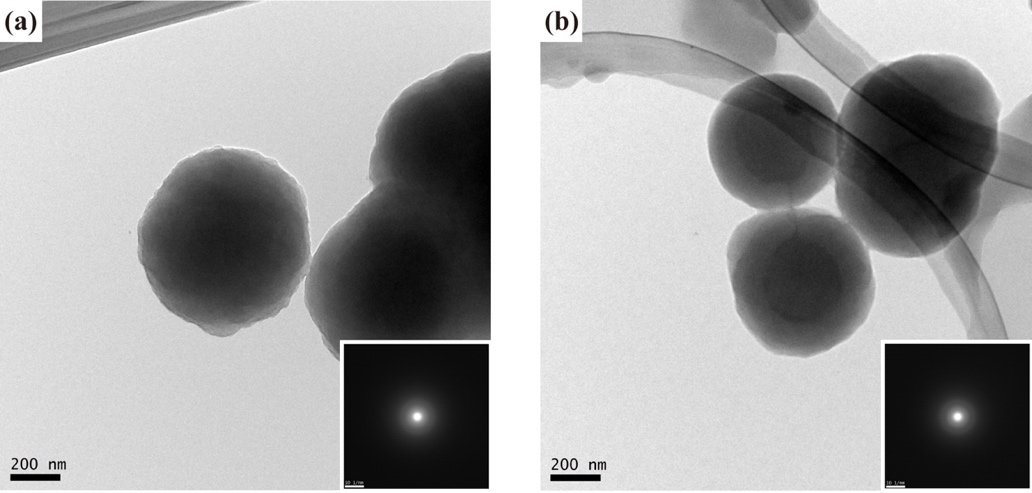


**Fig. S4 TEM images of particles composed of (6,6)CNBs and C_60_ molecules.** Selected area electron diffraction (SAED) patterns are also shown. (a) Particles before thermal treatment. (b) Particles after thermal treatment at 600 °C. The scale bars represent 200 nm.


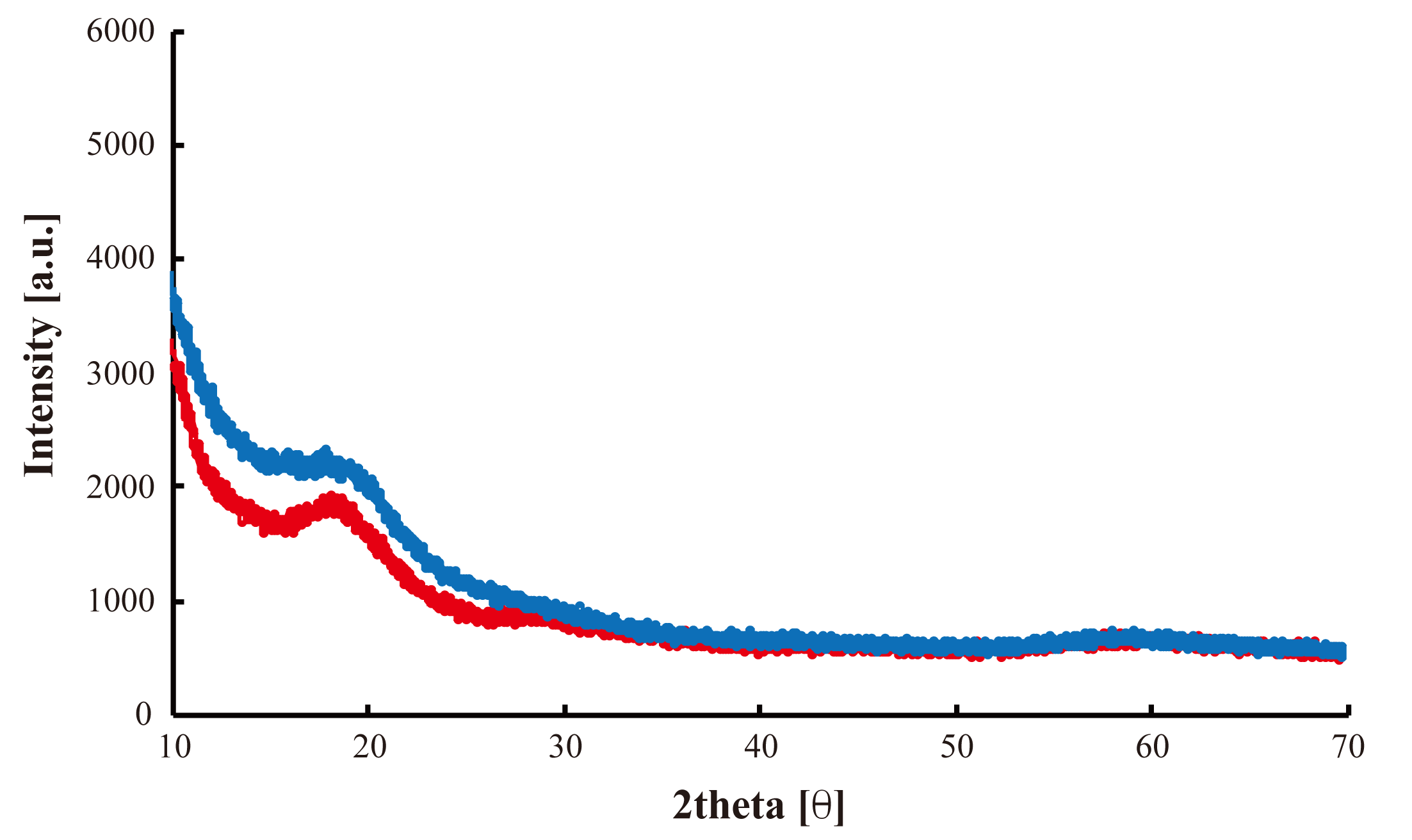


**
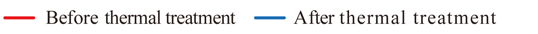
Fig. S5 XRD spectra of particles formed by compounds composed of (6,6)CNBs and C_60_ molecules.**


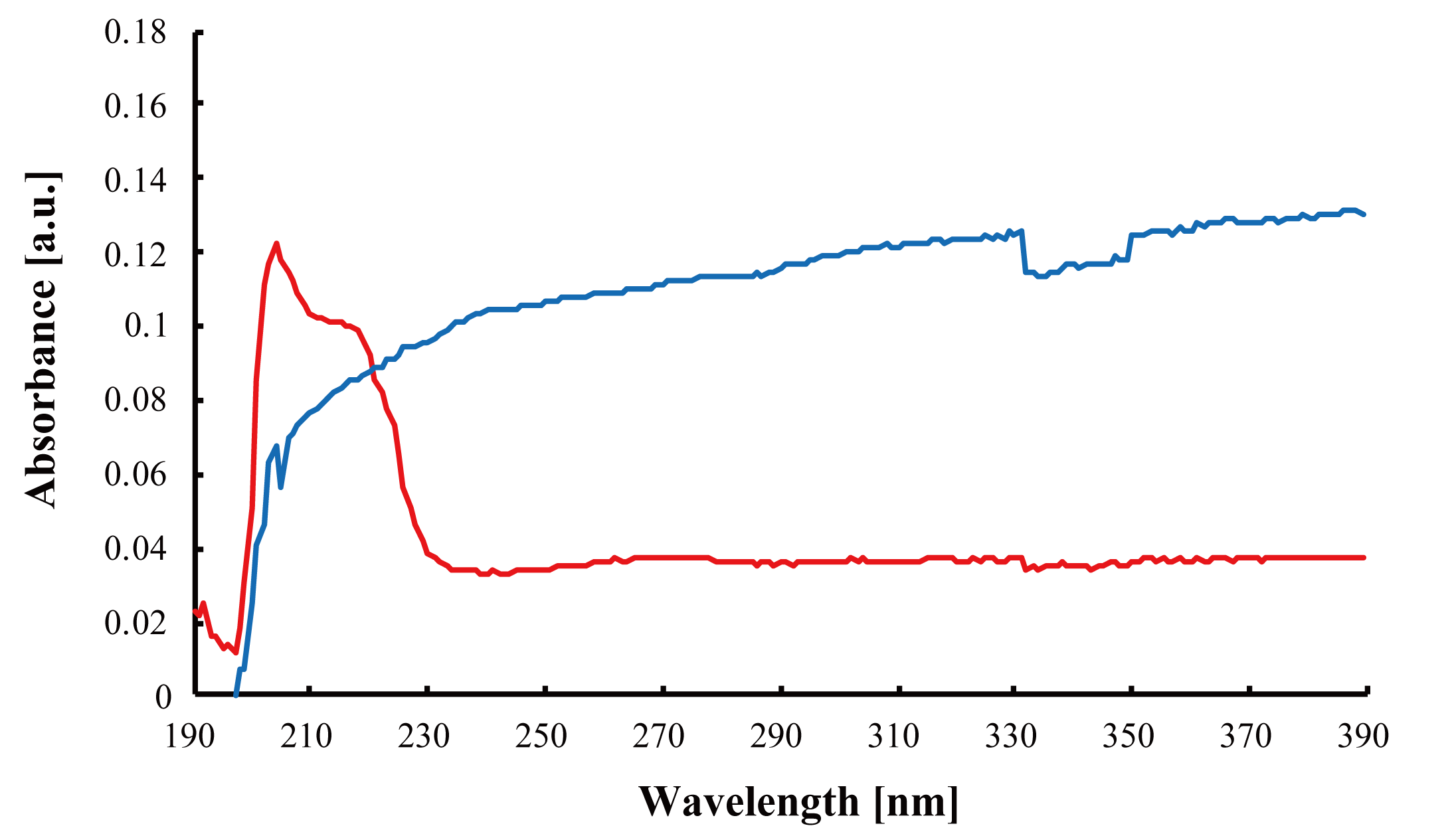


**Fig. S6 Absorption spectra by particles dispersed in ethanol.**


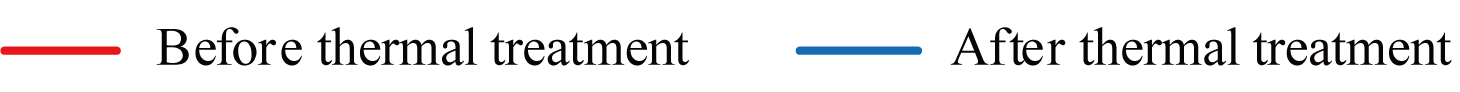


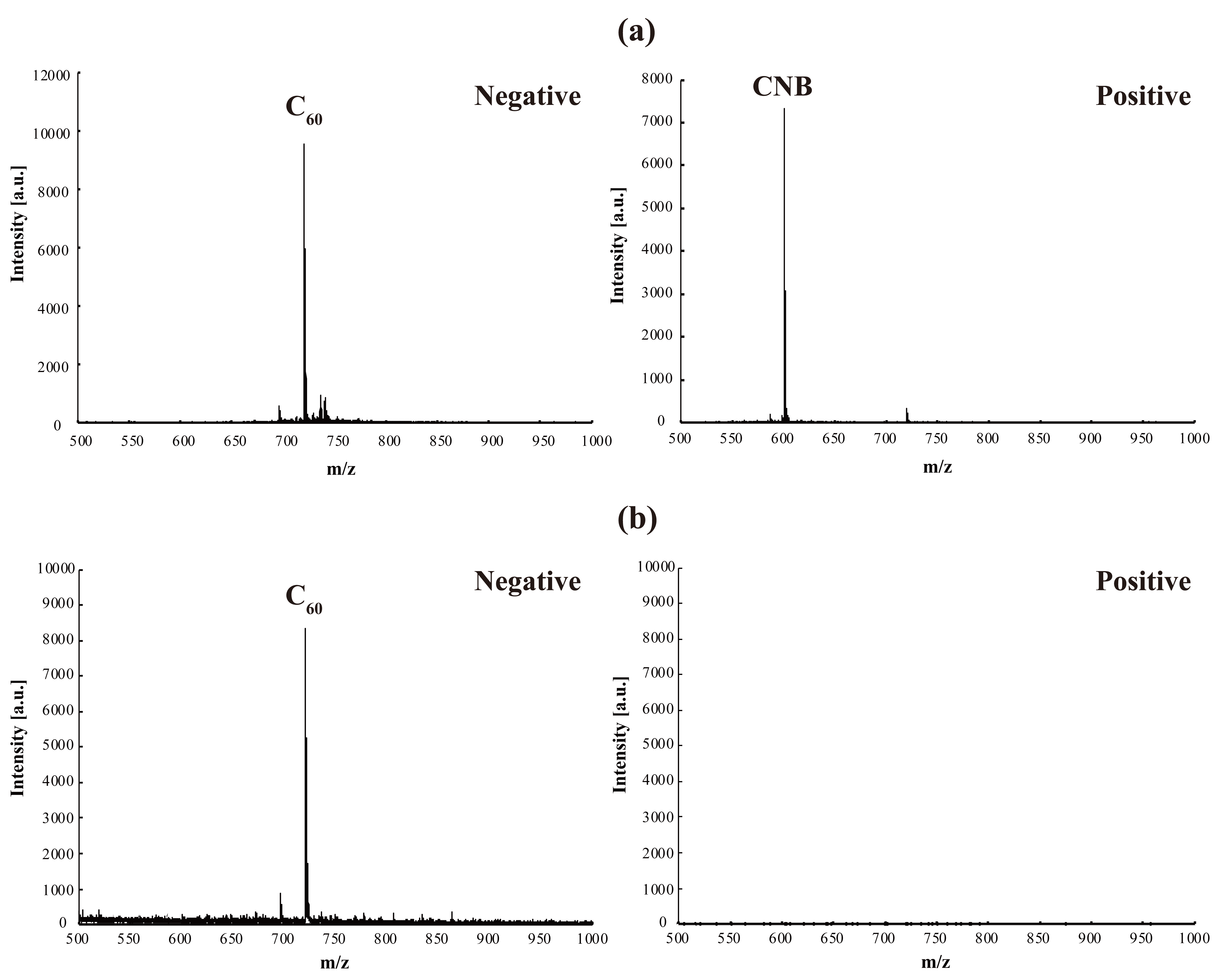


**Fig. S7 Mass spectra of the components forming particles.** (a) Before thermal treatment. (b) After thermal treatment at 600 °C.

Fig. S8 was changed as follows.


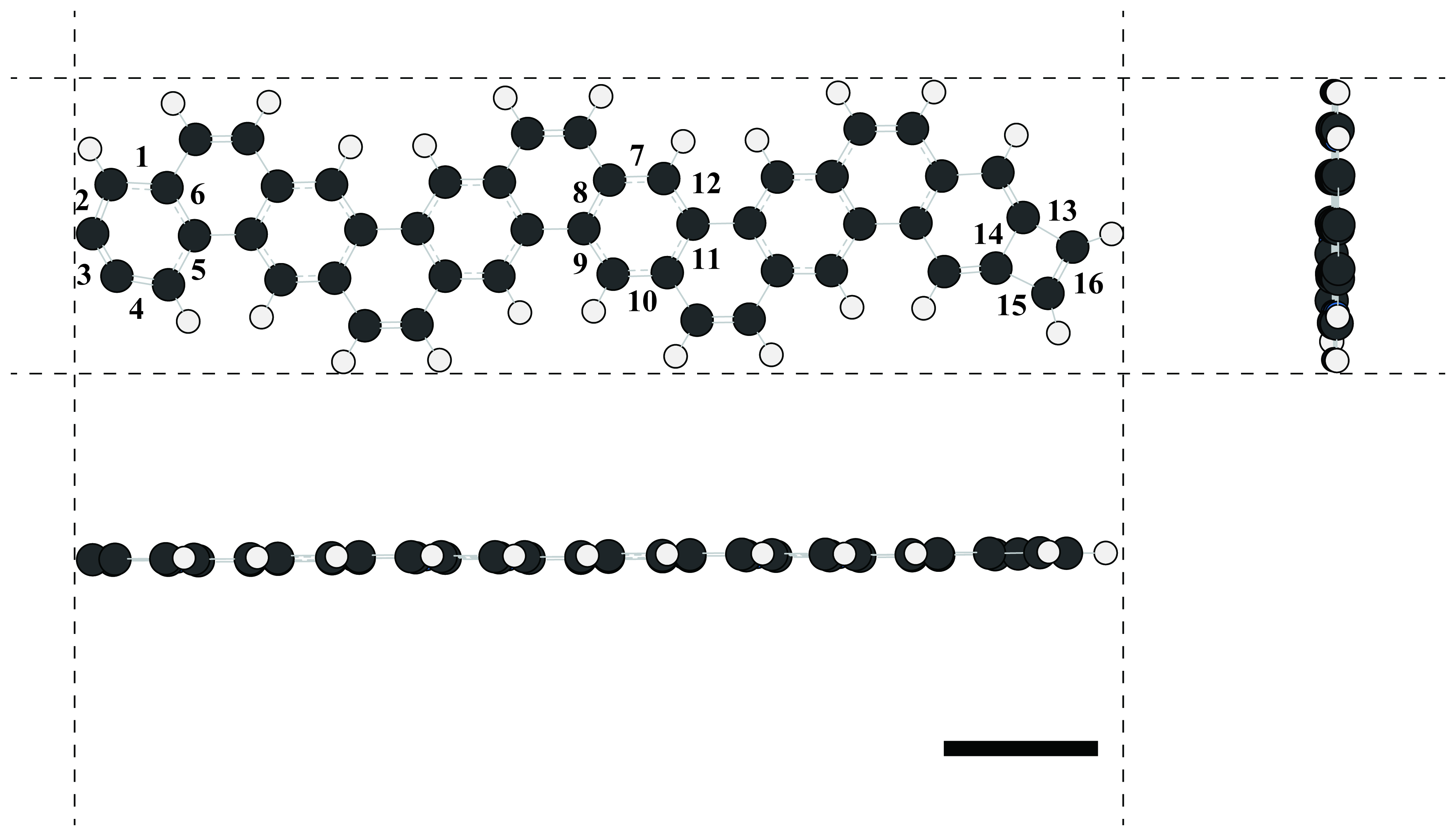


**(a)**

**(b)**

| **No.**  **indicated**  **in (a)** | **PM6 (nm)** | **DFT (nm)** |
| --- | --- | --- |
| 1 | 0.145 | 0.143 |
| 2 | 0.136 | 0.137 |
| 3 | 0.127 | 0.125 |
| 4 | 0.136 | 0.137 |
| 5 | 0.144 | 0.143 |
| 6 | 0.144 | 0.144 |
| 7 | 0.140 | 0.140 |
| 8 | 0.142 | 0.143 |
| 9 | 0.140 | 0.140 |
| 10 | 0.140 | 0.140 |
| 11 | 0.143 | 0.143 |
| 12 | 0.140 | 0.140 |
| 13 | 0.150 | 0.151 |
| 14 | 0.148 | 0.144 |
| 15 | 0.150 | 0.151 |
| 16 | 0.136 | 0.136 |

**Fig. S8 Carbon nano ribbon (CNR) calculated with PM6 and DFT.** (a) CNR calculated with the PM6 and DFT methods. Gray and white circles represent carbon and hydrogen atoms. A CNR terminated with a square is most stable in both cases of PM6 and DFT. The decomposition energy $E\equiv E\left( \mathrm{CNR} \right)-E\left( \mathrm{CNB} \right)=3.12 \mathrm{eV} and 0.065 eV/atom$ (PM6) and $1.92 \mathrm{eV}=0.04 eV/atom$ (DFT). The scale bar represents 0.4 nm. (b) Length between carbon atoms indicated in (a).


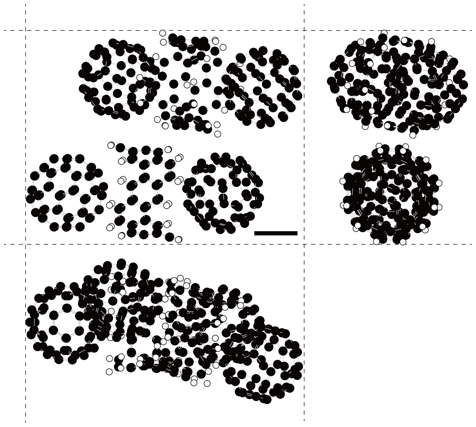

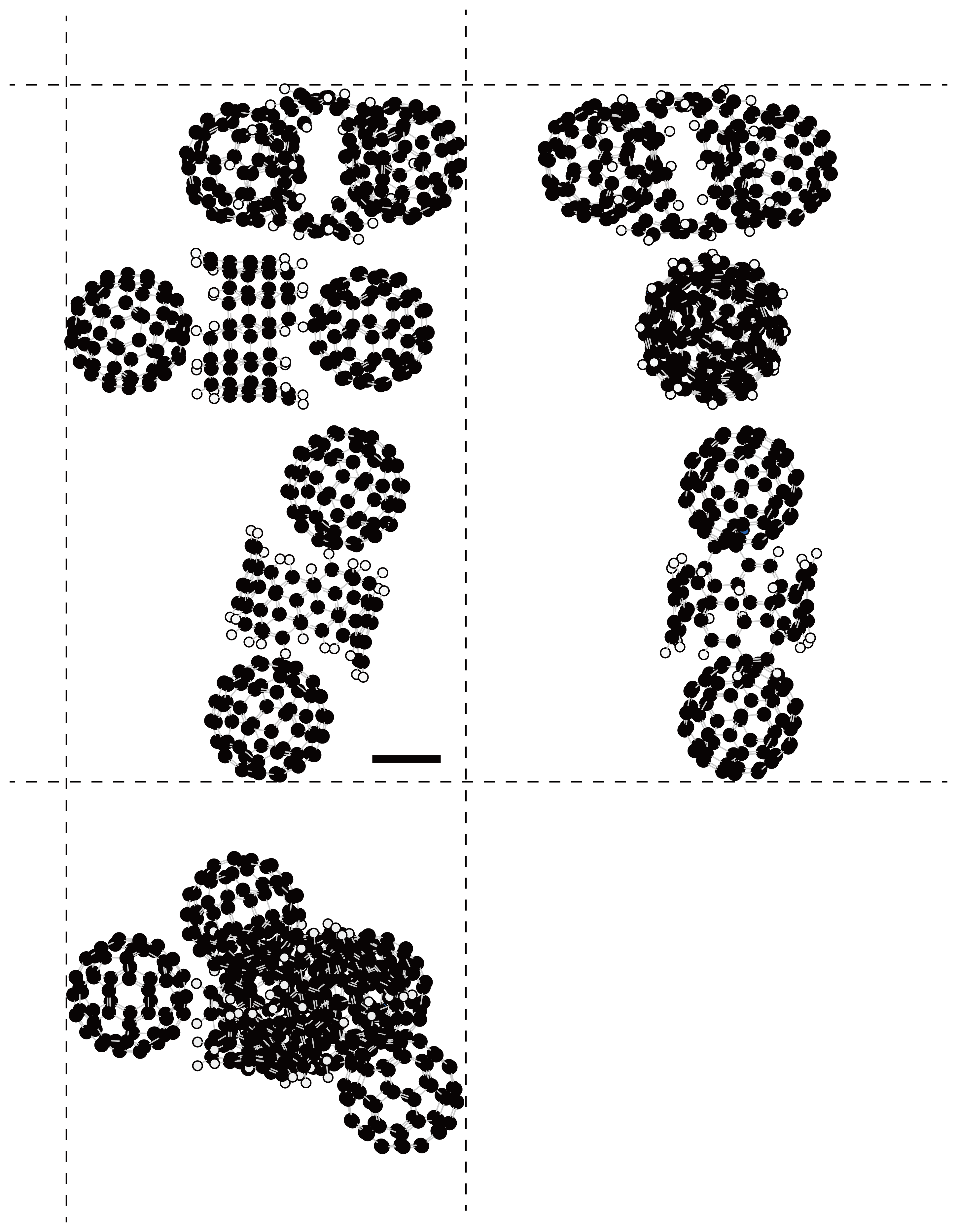


**(a)**

**(b)**

**Fig. S9 Configurations formed by compounds; C_60_-CNB-C_60_, obtained by PM6.** (a) Configuration formed by two compounds. The formation energy $E\equiv E\left( 2\times{(C}_{60}-\mathrm{CN}B-C_{60}) \right)-2\times E\left( \mathrm{CN}B \right)-4\times E(C_{60})=- 0.56 \mathrm{eV}$. (b) Configuration formed by three compounds. The formation energy $E\equiv E\left( 3\times{(C}_{60}-\mathrm{CN}B-C_{60}) \right)-3\times E\left( \mathrm{CN}B \right)-6\times E\left( C_{60} \right)=- 0.82 \mathrm{eV}$. The scale bars represent 0.4 nm.

**Video S1 (Video S1.mp4) Monodispersibility of the particles in distilled water.** The particles were thermally treated at 600 °C for 1 h and dispersed in distilled water.
